# Long-term Follow-up of Remnant Capsules After Implant Removal Without Capsulectomy: An Animal Model Study

**DOI:** 10.1093/asj/sjaf089

**Published:** 2025-06-16

**Authors:** Jaewoo Kim, Heewoong Yang, Jeongmok Cho, Ki Yong Hong, Hak Chang

## Abstract

**Background:**

Breast implantation induces fibrous capsule formation as a natural foreign body response. Although capsule-related complications often require capsulectomy, the need for capsulectomy during implant removal in patients with asymptomatic capsules remains unclear.

**Objectives:**

In this study, the authors aim to examine the capsule's degradation process over time and identify key cytokines involved in its remodeling.

**Methods:**

Nine 7-week-old Sprague–Dawley rats received custom 2.25 cm silicone cohesive gel implants subcutaneously on both dorsal sides. Capsule tissues were collected at implant removal (0M) and 3-month (3M) and 6-month (6M) intervals following implant removal without capsulectomy. Tissues were analyzed histologically utilizing hematoxylin and eosin and Masson's trichrome staining. Immunohistochemical staining for matrix metalloproteinase-9 (MMP9), CD31, α-smooth muscle actin (αSMA), Type I collagen (COL1), and CD68 was quantified for comparative analysis.

**Results:**

The 0M group showed robust capsule formation with dense collagen and myofibroblasts. Over time, the capsules thinned (315 µm at 0M, 194.8 µm at 3M, 136.8 µm at 6M; *P* < .01), became structurally disrupted, and merged with surrounding adipose tissue. Notably, MMP9 and CD31 levels increased significantly, indicating enhanced matrix turnover and vascularization. αSMA and COL1 declined initially before partially rebounding at 6M, whereas CD68 exhibited trends consistent with ongoing remodeling.

**Conclusions:**

The findings illustrate the natural degradation and remodeling of implant-induced capsules driven by specific cytokines and matrix markers. These insights may inform future guidelines on the necessity of capsulectomy during implant removal.

Breast implantation, whether for reconstruction following mastectomy or cosmetic enhancement, remains one of the most performed surgical procedures globally.^1,2^ The widespread adoption of breast augmentation has necessitated understanding the biological and clinical implications associated with implant placement.

Following the insertion of a breast implant, the body initiates a foreign body response characterized by the formation of a fibrous capsule around the implant. This encapsulation process is a natural aspect of wound healing and isolates the implant from surrounding tissues.^3^ The capsule formation around implanted foreign materials is driven by the body's immunological response. Upon implantation, fibrinogen and plasma proteins rapidly adhere to the surface of the device, a process known as biofouling. Macrophages subsequently bind to these protein-coated surfaces through specific receptors, fuse into multinucleated giant cells, and release transforming growth factor-β (TGF-β) and other inflammatory cytokines. These signaling molecules activate quiescent fibroblasts, transforming them into myofibroblasts that initiate procollagen synthesis through Smad signaling pathways. Secreted procollagen undergoes extracellular crosslinking, forming mature collagen and other extracellular matrix proteins. Over time, this results in the development of a dense, hypocellular fibrous capsule that is impermeable or hypopermeable to many substances, known as the capsule.^4-7^

The capsule consists of 3 distinct layers: closest to the implant, the inner cellular layer is rich in fibroblasts, T cells, and macrophages. Fibroblasts are essential for collagen synthesis, whereas macrophages and T cells play roles in immune response and tissue remodeling. Surrounding the inner layer is a middle layer composed of vascularized loose connective tissue. This layer supports nutrient delivery, oxygen exchange, and immune cell movement, all of which are essential for maintaining cellular activity and tissue health. The outermost layer consists of dense connective tissue with additional vascularization. It provides mechanical strength and stability to the capsule, with dense collagen fibers creating a robust extracellular matrix that restricts permeability. Together, these layers ensure the capsule's integrity.^8-10^

The formation of fibrous capsules represents an evolutionarily conserved protective mechanism through which the body isolates substances or pathogens resistant to phagocytic elimination. This containment strategy effectively limits potential inflammatory responses and tissue damage caused by persistent foreign materials. The encapsulation process reflects a fundamental biological adaptation aimed at restoring homeostasis when confronted with unresolvable foreign bodies. Although this defensive mechanism prevents systemic dissemination of potentially harmful materials, it simultaneously creates challenges for the functionality of medical implants by potentially restricting tissue integration and impeding therapeutic agent diffusion from implanted devices.^11,12^

Although mostly asymptomatic, abnormal capsule formation has been associated with a condition known as capsular contracture. Although the exact cause is unclear, the incidence of capsular contracture has been linked to radiotherapy, infection, or the type of implant surface.^13-15^ Therefore, in cases where reoperation is required owing to symptoms regarding capsular contracture, the Breast Surgery Collaborative Community (BSCC) guidelines, developed by a team of physician representatives of the American Society of Plastic Surgeons (ASPS) and The Aesthetic Society, established in 2024, advocate for a full capsulectomy. Nonetheless, some uncertainty remains regarding whether capsulectomy should be performed in cases requiring simple removal or exchange of the breast implant without clear signs of abnormal capsule formation. The guidelines suggest that patients with suspected breast implant-associated cancer should undergo an en bloc capsulectomy. However, no consensus has been reached regarding asymptomatic cases. The BSCC guidelines suggest a full-depth discussion and leave the final choice to the patient.^16^

In a systemic cohort review in 2024, McGuire et al suggested that patients with breast implant illness (BII) reported significantly improved symptoms 6 months after explantation, without capsulectomy. They also reported that the symptom alleviation is not significantly different than that in the patients who received partial or total capsulectomy.^17^ In another study, the authors suggest that given the diverse inflammatory findings and microbial growths in BII, the use of total capsulectomy with implant removal is advocated.^18^ However, partial or total capsulectomy increases risks of postoperative complications, such as hematoma or infection.^19^ Thus, the standard guidelines for capsulectomy remain unclear.

Similarly, not much is known about the natural course of the capsule when left inside the breast pocket. No reports in MEDLINE or PubMed database (US National Library of Medicine, Bethesda, MD) discuss long-term changes in the leftover capsule after removal of the silicone implant. Therefore, the aim of the authors of this study was to simulate the internal microenvironment of the silicone breast implant and its surroundings in a rat model and understand the course of the remnant breast capsule when left in vivo.

## METHODS

### Animal Model and Ethical Approval

This animal study was conducted between March 2023 and January 2024. The experimental protocol was approved by the Institutional Animal Care and Use Committee (IACUC) of Seoul National University Hospital (IACUC number 23-0204-S1A1). Nine 7-week-old Sprague–Dawley rats (Koatech, Pyeongtaek, Korea) were obtained and housed in pairs under controlled conditions, including a 12-h artificial light/dark cycle, regulated temperature, and ad libitum access to food and water. Animal care was conducted in accordance with institutional and national ethical guidelines to minimize pain and distress.

### Implant Placement Procedure

Custom-made smooth-surfaced, round silicone cohesive gel implants (HansBioMed, Seoul, Korea), designed to mimic the shape of a breast implant, were used. The implants were 3 mL in volume and 2.25 cm in diameter. The implants were inserted subcutaneously on the dorsal side of each rat, with 1 implant placed on the left and the other on the right. All procedures were performed under anesthesia utilizing 3% inhaled isoflurane (KyongBo Pharmaceutical, Asan, Korea). After shaving and disinfecting the surgical site, a 2 cm incision was made, followed by subcutaneous dissection to create a pocket for the implant. The implant was placed, and the wound was closed utilizing 4-0 Vicryl (Johnson & Johnson MedTech, New Brunswick, NJ) sutures, and the implant insertion site was tagged with 5-0 Ethilon (Johnson & Johnson MedTech) sutures. Owing to the invasiveness of the procedure and the difficulty of stitch removal, absorbable sutures were used. Although the implant was not sutured in place, it was positioned securely to prevent movement. The procedure was then repeated on the opposite side to ensure that each rat carried 2 implants in separate subcutaneous pockets. All procedures were performed aseptically.

### Implant Removal and Sample Collection

**Figure 1.**
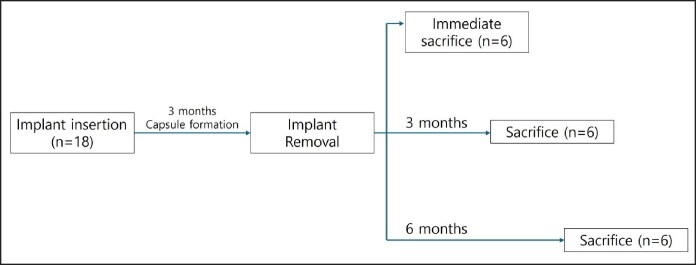
The schematic diagram of the experiment.

The implants remained in place for 3 months to allow for physiological capsule formation. Capsule formation was confirmed utilizing ultrasound and micro-CT before implant removal. To facilitate accurate identification of capsule location, skin marking with nylon sutures was performed before implant removal. The implants were removed through the original incisions, and tissue samples were collected at 3 time points. In the immediate removal group (0M group), 3 rats were sacrificed immediately after implant removal to obtain fresh capsule tissue. In the 3-month postremoval group (3M group), 3 rats were sacrificed 3 months after implant removal. In the 6-month postremoval group (6M group), the remaining 3 rats were sacrificed 6 months after implant removal (Figure 1).

### Histological and Immunohistochemical Analysis

Capsule specimens were meticulously harvested utilizing an en bloc technique to minimize mechanical disruption and preserve tissue integrity. Following excision, each specimen was carefully bisected and oriented to maintain anatomical landmarks, ensuring consistency across samples. Collected tissue samples were fixed overnight in 4% paraformaldehyde (Becton Dickinson, Franklin Lakes, NJ), dehydrated, and embedded in paraffin. Sectioning was performed to obtain representative tissue slices from the central, superficial, and deep regions of the capsule, thereby capturing the full histological spectrum of the fibrous envelope. Multiple sections from each region were subsequently analyzed to provide a comprehensive assessment of regional variation in capsule morphology and cellular composition.

Histological analysis was performed utilizing hematoxylin and eosin staining to assess cellular composition and Masson's trichrome staining to evaluate collagen structure. Capsule thickness was measured at 8 randomly selected points per slide. Inflammation was assessed utilizing a 5-point scale (Grade 0: none; Grade 1: 0%-20% of inflammatory cells in 5 randomly selected high-power fields; Grade 2: 20%-40%, Grade 3: 40%-60%; Grade 4: 60%-80%; Grade 5: 80%-100%), with evaluations conducted by 2 independent observers.^20^

Immunohistochemistry (IHC) staining was performed on selected samples to analyze key markers involved in capsule formation and degradation, including matrix metalloproteinase-9 (MMP9) for extracellular matrix remodeling, CD31 for endothelial marker of vascularization, α-smooth muscle actin (αSMA) for myofibroblast activity, Type I collagen (COL1) for structural integrity of the capsule, and CD68 for macrophage presence and immune response. Signal intensity was analyzed by ImageJ (National Institutes of Health, Bethesda, MD) and Python (Python Software Foundation, Beaverton, OR) software.

### Statistical Analysis

Capsule thickness and IHC signal intensity were statistically compared across time points (0M, 3M, and 6M) by performing the Kruskal–Wallis test because of the small sample size and non-normal distribution. Statistical analysis was performed utilizing Python software, and graphical representations were generated with SigmaPlot (Grafiti LLC, Palo Alto, CA). Statistical significance was determined as *P < .05, **P < .01, and ***P < .001.

## RESULTS

**Figure 2.**
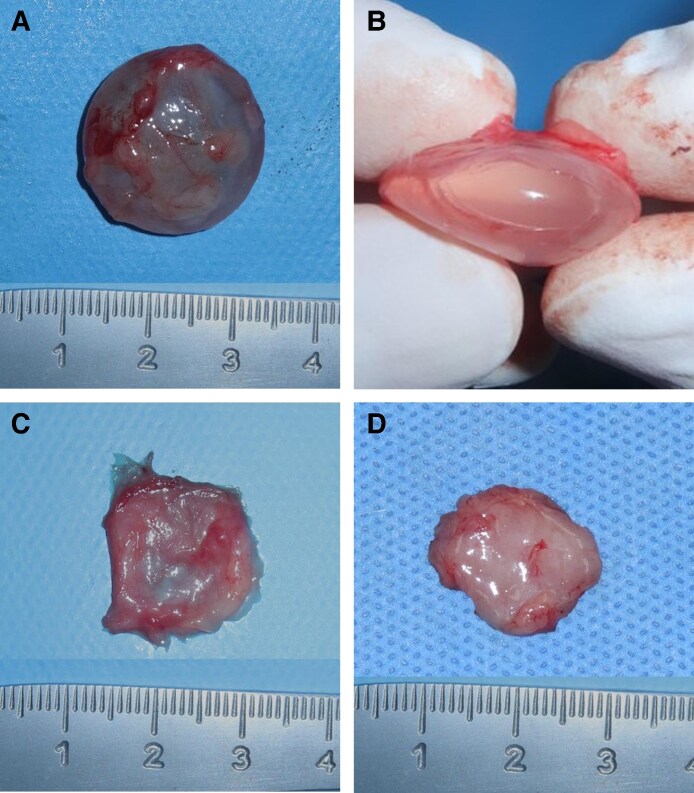
Gross photograph of retrieved capsules. (A) Robust capsule wrapping around the experimental silicone implant. (B) Explantation of silicone implant from wrapping capsule. (C) Gross photograph of fresh capsule from the 0M group. (D) Gross photograph of degraded capsule from 6M group.

During the experimental period, there were no instances of mortality or adverse events, such as infection, hematoma, implant rupture, or malposition, throughout the duration of the study. Gross inspection of the harvested specimens at the 0M time point demonstrated robust capsule formation. The capsule measured ∼2.1 × 2.2 cm in surface dimensions, with a thickness of <0.1 cm. However, in the 3M and 6M groups, the capsules had undergone significant degradation and were not readily distinguishable from the surrounding tissue. Nevertheless, portions of tissue suggestive of remnant capsules were identified. Therefore, the surrounding subcutaneous tissue was harvested together, and additional microscopic analysis was performed to confirm the presence of residual capsule structures (Figure 2).

**Figure 3.**
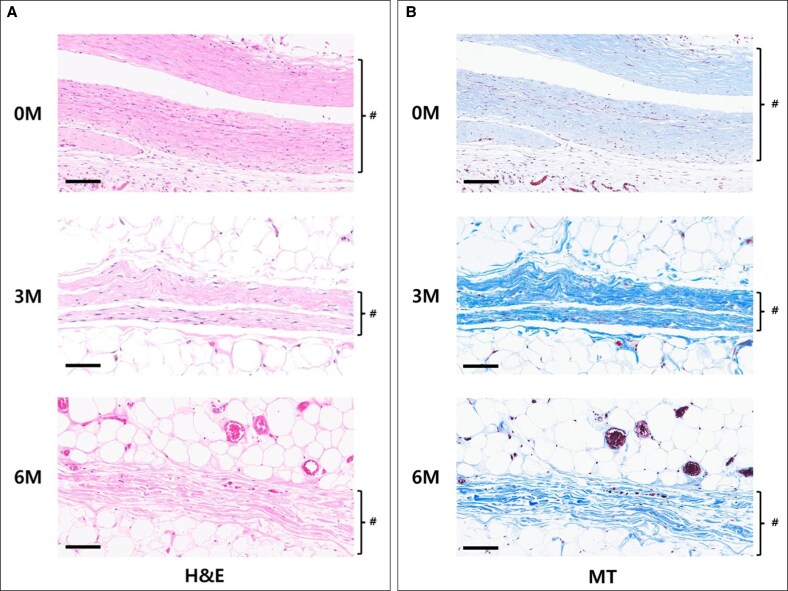
(A) The representative slides of hematoxylin and eosin staining, showing capsule degradation. The capsule is marked in # (scale bar = 100 μm). (B) The representative slides of Masson's Trichrome staining, showing capsule degradation. The capsule is marked in # (scale bar = 100 μm).

**Figure 4.**
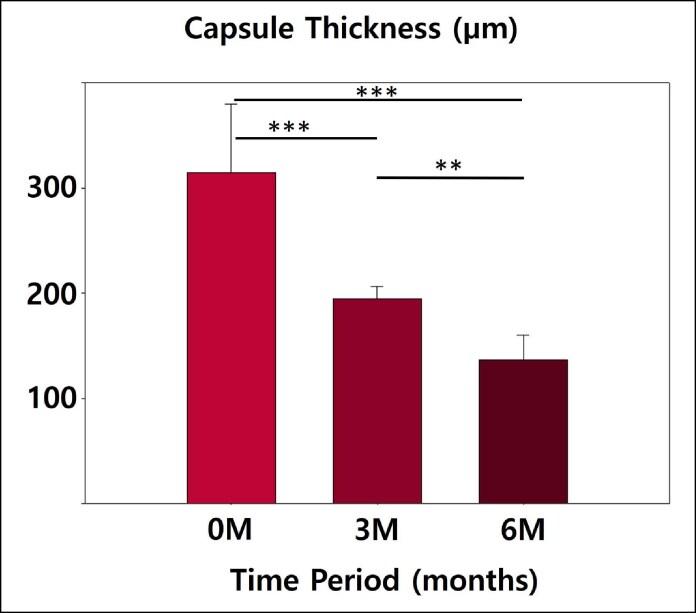
The median capsule thickness after explantation (**P < .01, ***P < .001).

Histological analysis revealed fresh and robust capsule formation in the 0M group, characterized by dense and organized collagen and myofibroblast structures. However, significant degradation was observed in the 3M and 6M groups in chronological order. In these groups, the capsules exhibited thinning, loss of structural integrity, and disrupted architecture. No evidence of silicone leakage or residual silicone was observed in any of the capsule specimens, and all explanted implants were confirmed to be intact upon removal. Furthermore, increased integration with the surrounding soft tissue, predominantly composed of adipocytes, was observed (Figure 3). The median thickness of the fresh capsule in the 0M group was 315 µm, with an interquartile range (IQR) of 64.6 µm. At the 3-month follow-up (3M group), the median capsule thickness decreased to 194.8 µm (IQR 31.34 µm). By the 6-month follow-up (6M group), further reduction in capsule thickness was noted, with a median value of 136.8 µm and an IQR of 41.65 µm. Statistical analysis of the dataset demonstrated significant differences across time points (*P* < .01; Figure 4).

The inflammation index, introduced by a 5-point scaling system to evaluate the degree of inflammation, was noted by 2 independent researchers and showed no significant differences (*P* > .9). Inflammatory responses were minimal in all samples, with <20% inflammatory cells, indicating limited immune-mediated reactions regardless of the time point after capsule formation.

**Figure 5.**
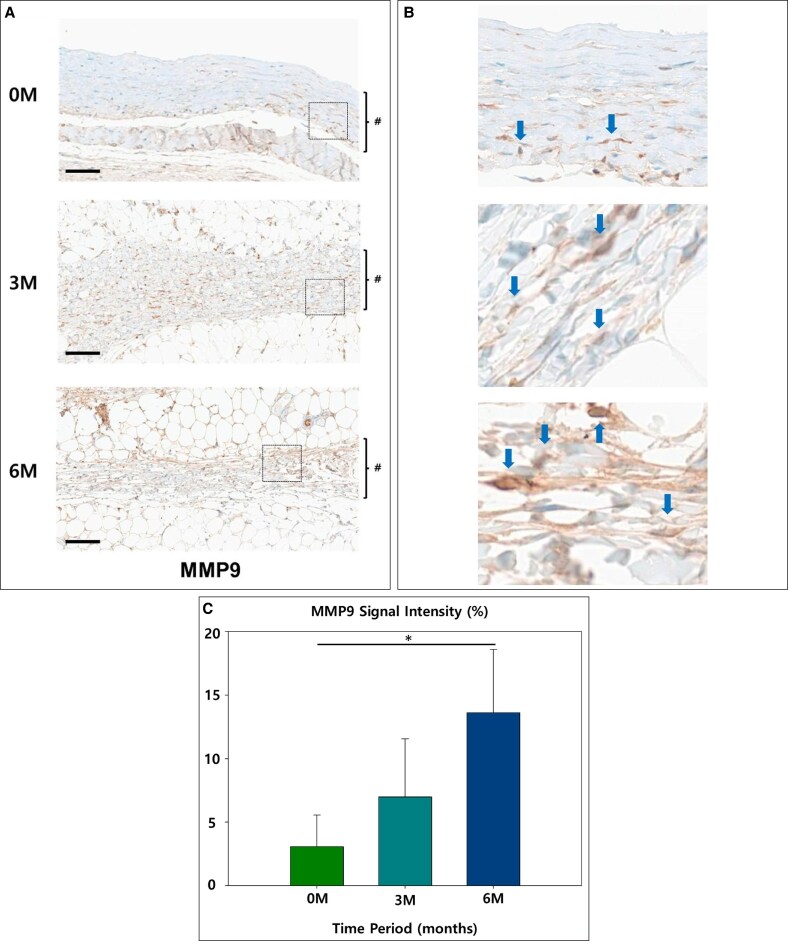
(A) The representative slides of immunohistochemistry of matrix metalloproteinase-9 (MMP9): the capsule is marked in # (scale bar = 100 μm). (B) 400× magnification view: the stained cells are indicated by blue arrows. (C) Graph of relative signal strength (*P < .05).

**Figure 6.**
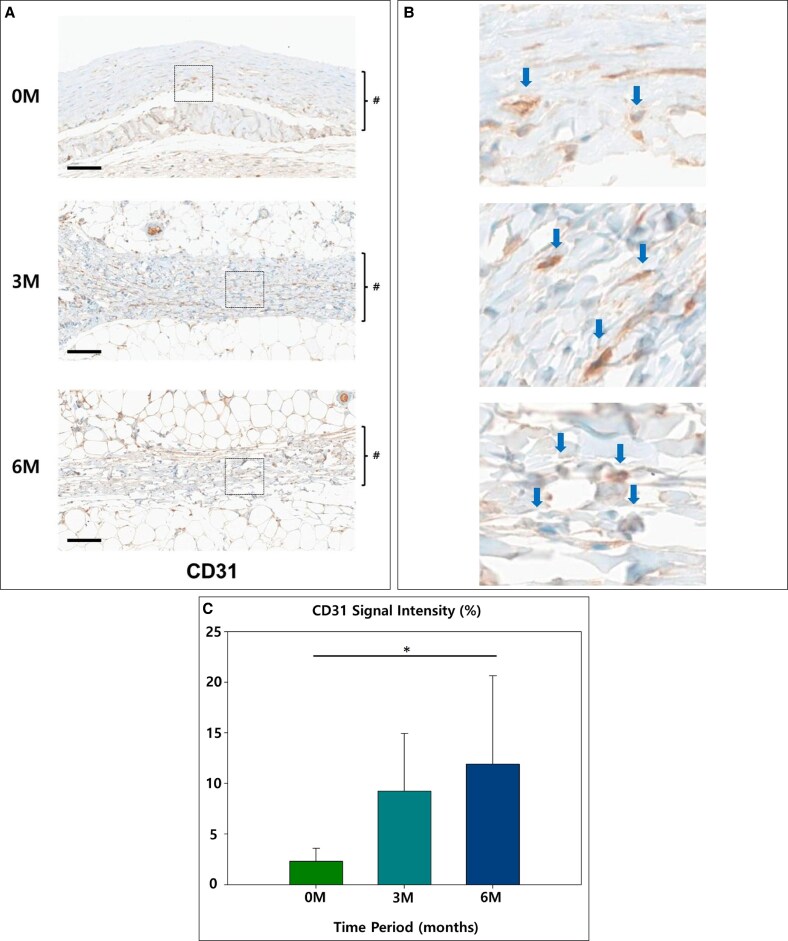
(A) The representative slides of immunohistochemistry of CD31: the capsule is marked in # (scale bar = 100 μm). (B) 400 × magnification view: the stained cells are indicated by blue arrows. (C) Graph of relative signal strength (*P < .05).

Markers associated with vascularization and collagen breakdown showed the strongest inverse relationship with time. MMP9, which reflects extracellular matrix turnover, started at 2.58%, suggesting early matrix activity. However, it increased significantly to 9.35% at 3 months, indicating active matrix remodeling, and further increased to 13.63% at 6 months, highlighting sustained extracellular matrix activity over time (*P* = .04; Figure 5). CD31, a marker of endothelial activity, was relatively low at 2.32% in the 0M group. However, it showed a significant increase to 9.26% at 3 months, reflecting enhanced vascularization, and continued rising to 11.93% at 6 months, indicating vascular stabilization (*P* = .04; Figure 6).

**Figure 7.**
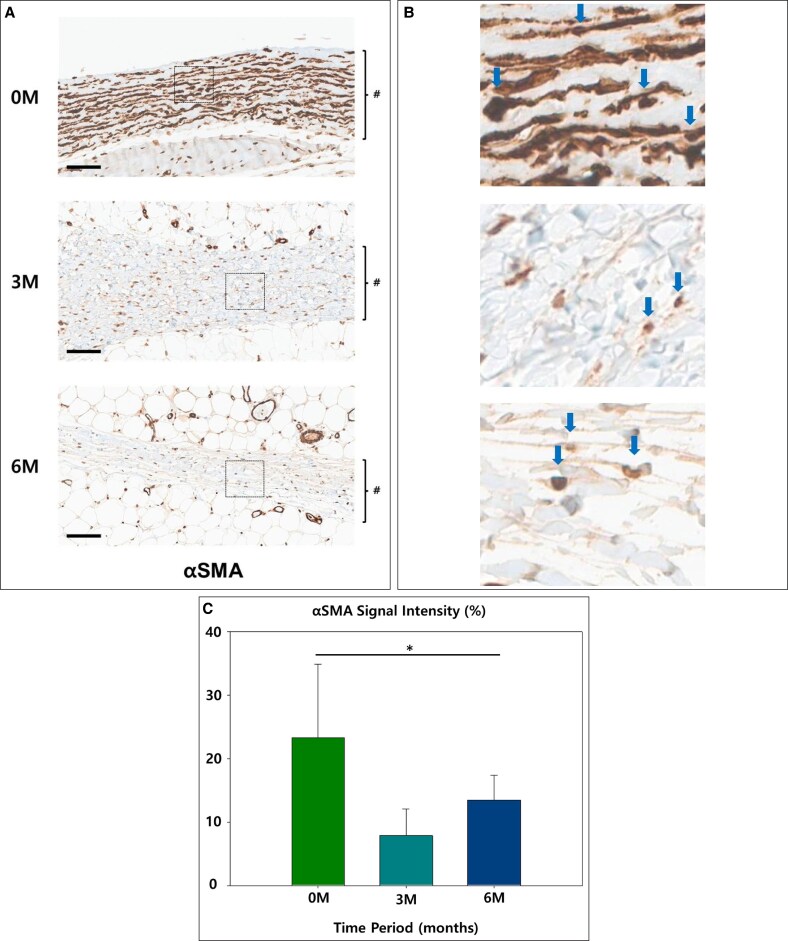
(A) The representative slides of immunohistochemistry of α-smooth muscle actin (αSMA): the capsule is marked in # (scale bar = 100 μm). (B) 400× magnification view: the stained cells are indicated by blue arrows. (C) Graph of relative signal strength (*P < .05).

**Figure 8.**
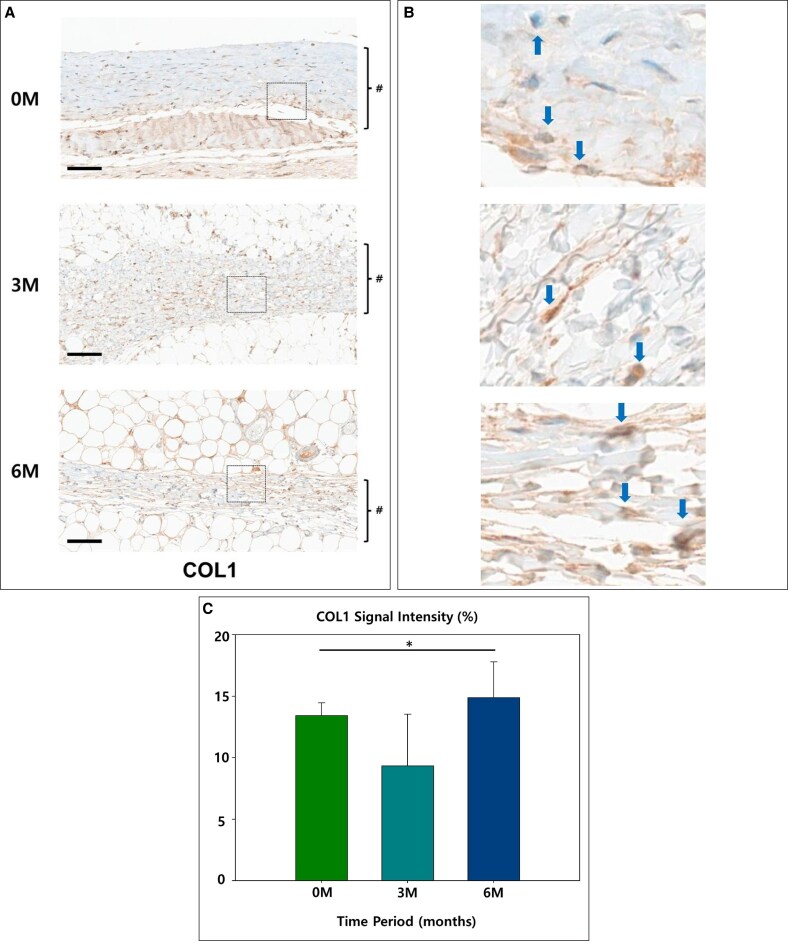
(A) The representative slides of immunohistochemistry of Type I collagen (COL1): the capsule is marked in # (scale bar = 100 μm). (B) 400× magnification view: the stained cells are indicated by blue arrows. (C) Graph of relative signal strength (*P < .05).

Factors associated with capsule formation showed somewhat mixed relationships with time. αSMA started at 23.35% in the 0M group, indicating high myofibroblast activity. It sharply decreased to 7.87% in the 3M group, reflecting a decline in myofibroblast-driven repair. However, in the 6M group, it slightly increased to 13.49%, suggesting a secondary phase of tissue restructuring (*P* = .02; Figure 7). COL1 was initially 13.44%, reflecting active collagen deposition. It decreased to 9.35% in the 3M group, indicating a reduction in early matrix deposition, but increased to 14.90% in the 6M group, suggesting ongoing extracellular matrix remodeling (*P* = .04; Figure 8).

**Figure 9.**
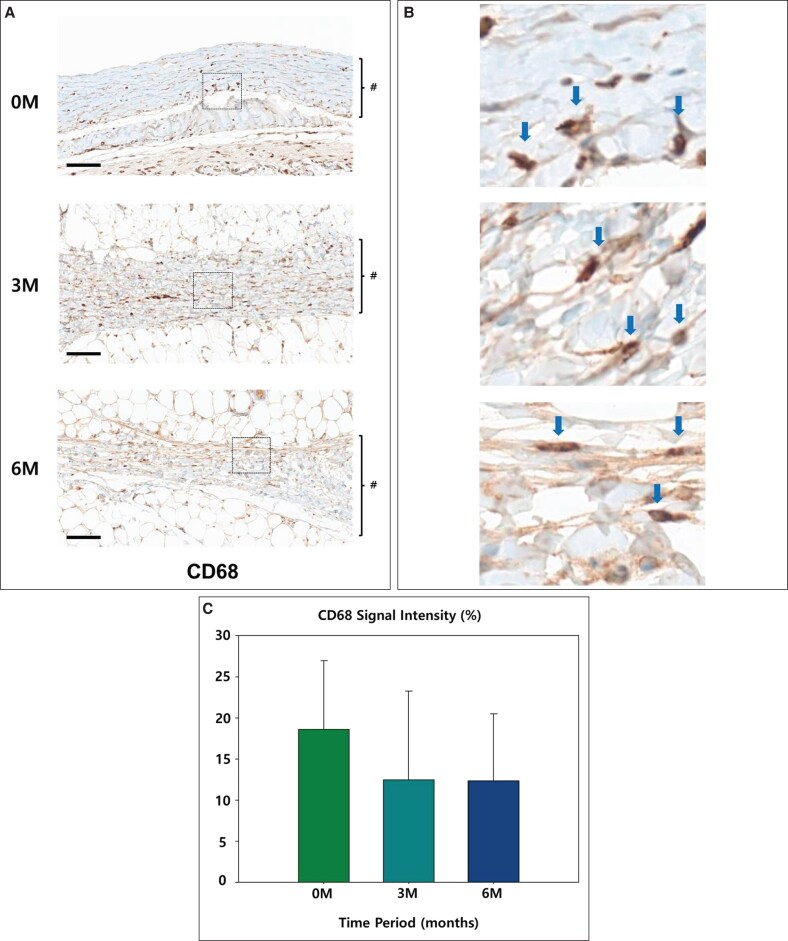
(A) The representative slides of immunohistochemistry of CD68: the capsule is marked in # (scale bar = 100 μm). (B) 400× magnification view: The stained cells are indicated by blue arrows. (C) Graph of relative signal strength.

CD68, a marker of macrophage presence, started at 18.64%, indicating notable inflammatory activity. It decreased to 12.48% in the 3M group, reflecting a reduction in inflammation, and remained relatively stable at 12.36% in the 6M group, suggesting persistent low-level macrophage activity (*P* = .37; Figure 9).

## DISCUSSION

Breast implant placement remains one of the most prevalent foreign body insertion procedures worldwide, primarily performed for aesthetic augmentation or postmastectomy reconstruction. The formation of peri-implant capsules occurs through complex immune-mediated chain reactions involving fibroblast activation and collagen deposition. Once established, complete capsule removal presents significant technical challenges, often requiring extended operative time and substantial healthcare resources. Moreover, partial or total capsulectomy carries inherent risks, with a reported 47% increase in major complications (2.8% vs 1.9%) and a 78% higher hematoma incidence (1.6% vs 0.9%) compared with implant removal without capsule excision.^19^ In addition, the risk of seroma formation is also higher in capsulectomy procedures than in removal without capsulectomy.^21^ Additionally, capsulectomy is more commonly associated with breast tissue thinning and larger incision sizes than simple explantation.

These risks contribute to the frequent clinical decision to retain capsules during explantation procedures. However, persistent capsules may lead to clinically significant sequelae, most notably idiopathic capsular contracture, which can cause pain, breast deformity, and restricted upper-extremity range of motion. Rare but serious complications include late-onset calcification, particularly in implants retained for >10 years, and emerging yet rare reports of capsular-associated malignancies, for example, the formation of spiculate masses or densely calcified capsules appearing on mammography and MRIs, according to a series of case reports during the late 1990s to early 2000s and a meta-analysis in 2015.^22-26^

Although capsulectomy is common for addressing periprosthetic problems such as capsular contracture or implant malposition, it is not a universally established standard of care in routine explantation. Recent consensus statements and patient safety advisories emphasize that the absolute indication for en bloc capsulectomy is limited to cases with an established or suspected breast implant-associated malignancy, such as breast implant-associated anaplastic large cell lymphoma, after appropriate medical evaluation.^16^ Current clinical practices lack standardized guidelines for capsule management, highlighting the critical need for evidence-based protocols. This experimental study investigated longitudinal histological changes in retained capsules following uncomplicated explantation in an animal model, aiming to characterize natural progression patterns and inform clinical decision making.

Even in procedures other than implant removal, the capsule may be left intact during breast revision surgery. Capsular contracture is classified by the Baker system, and, although capsulectomy is often performed for Grades 3 and 4, it is typically unnecessary for Grades 1 and 2.^27^ In such cases, pocket enlargement or lowering of a high-riding implant can be achieved with capsulotomy. Conversely, if pocket reduction is needed—such as for downsizing or correcting bottoming out or lateral displacement—capsulorrhaphy is preferred.^28^ Especially when the capsule is thin, capsulorrhaphy is favored over capsulectomy to reduce risk, and the capsule is left behind in the revised pocket. Even in some Grade 3 or Grade 4 cases, the capsule may be preserved. A frequently used approach involves placing the implant in a neoplane without removing the capsule, thereby avoiding complications and securing the implant in fresh tissue.^29^

To the best of our knowledge, our experiment is the first to scientifically predict and prove the partial, if not total, breakdown and resorption of capsules when left in the body, even when showing a noneventful course. In some cases where silicone leakage is noted, a residual capsule has also been detected on MRIs.^25^ In a normally formed sound capsule, after removal of the foreign body, the capsule slowly starts to degrade and emits cytokines such as MMP9 and VEGF-R for breakdown of extracellular matrix and fibrous tissue.^30^ However, further studies are needed to understand the factors influencing capsule persistence vs degradation in vivo, particularly in long-term follow-up.

The development of fibrous capsules in small animal models has been well-documented across studies investigating implant-related foreign body responses, as demonstrated by Fischer et al.^31^ In our study, capsule formation and degradation was observed over ∼9 months, representing a notably extended duration for experimentation in a rat model. The exceptionally long experimental period for a rat study is noteworthy, because it is likely allowed for a more accurate replication of the processes of capsule formation and degradation occurring within the human body.

Through immunohistochemical analysis, the factors predominantly involved in the capsule breakdown process during specific time points could be identified. COL1 and myofibroblasts are well-known structural components of the fibrous capsule, and macrophages control collagen accumulation.^32-34^ The decrease and subsequent gradual increase of αSMA and COL1, as well as the gradual increase of CD31 and MMP9, indicate the occurrence of breakdown and remodeling, considering the roles of each factor. CD68, which assesses monocytes and macrophages, remained relatively consistent and showed lower statistical significance, but a gradual decrease over time was also observed.

The reasons for the decrease in αSMA and COL1 levels in the 3M group are not entirely clear. This reduction may represent a transient phenomenon during the remodeling process or could be attributed to variations arising from the limited sample size. The increase in αSMA and COL1 may also be attributed to the relative rapidness of muscle degradation compared with collagen degradation, misrepresenting the percentage of collagen deposition in the 6M group.^35,36^ Given the steady increase of CD31 and MMP9, the decrease is more likely associated with a temporary decrease in the tissue remodeling process. However, further investigation is warranted, such as observing additional time points or conducting studies with larger sample sizes, to provide a more detailed understanding of these findings.

One of the limitations of this study is the relatively small sample size, which may affect the generalizability of the findings. Additionally, the implants were placed in the dorsum rather than in glandular tissue, which may not fully replicate the physiological conditions, such as hormonal cycles and specific immune responses, introducing potential variability in the experimental outcomes. Another limitation of this study is the use of an animal model. Although valuable for initial investigations, extrapolating findings from animal models to human physiology can be challenging because of inherent interspecies differences in capsule structure and function. Furthermore, current methodologies for studying human capsules in vivo present considerable limitations, including invasiveness, thereby hindering the ability to fully understand capsule behavior and responses to therapeutic interventions in humans. The study could also have benefited from advanced imaging modalities, such as MRI, to assess capsule thickness and degradation over time. Studies have discussed capsule thickness without animal sacrifice, utilizing noninvasive MRI with 4.7 or 7 T high-resolution imaging.^31,37^ Owing to the lack of an experimental MRI system, this study did not include MRI imaging. Incorporating such imaging techniques in future studies could provide more quantitative data on capsule remodeling and persistence. Finally, targeted investigations are needed to examine abnormal leftover capsules in the body, particularly those influenced by specific conditions that could mimic more precise clinical settings. For instance, the authors could explore the effects of electrocautery, adapting techniques such as popcorn capsulorraphy, or the role of radiation in inducing artificial capsular contracture. These approaches could provide valuable insights into the mechanisms underlying abnormal capsule persistence or degradation, which are crucial for optimizing clinical management strategies.

## CONCLUSIONS

Our study shows evidence of degradation of the physiologic capsule in animal models and the main cytokines involved in the process. Our findings also depict the changes in immunohistochemical markers that are critically involved in the capsule maturation and degradation process over a prolonged period. Further studies may provide more information about capsule remodeling and possibly help establish a guideline for performing capsulectomy.
